# Awareness and Knowledge of Antimicrobial Resistance, Antimicrobial Stewardship and Barriers to Implementing Antimicrobial Susceptibility Testing among Medical Laboratory Scientists in Nigeria: A Cross-Sectional Study

**DOI:** 10.3390/antibiotics12050815

**Published:** 2023-04-26

**Authors:** Sheng Huang, Ukpai A. Eze

**Affiliations:** 1 School of Life Sciences, Faculty of Health and Life Sciences, Coventry University, Coventry CV1 5FB, UK; 2 Leicester School of Allied Health Sciences, Faculty of Health and Life Sciences, De Montfort University, Leicester LE1 9BH, UK

**Keywords:** antibiotics, antimicrobial resistance, antimicrobial stewardship, diagnostic stewardship, antibiotic usage, medical laboratory scientists, antimicrobial susceptibility test, bacterial infection, infection prevention and control, Nigeria

## Abstract

**Background:** Antimicrobial resistance (AMR) is now considered one of the greatest global health threats. This is further compounded by a lack of new antibiotics in development. Antimicrobial stewardship programmes can improve and optimize the use of antibiotics, thereby increasing the cure rates of antibiotic treatment and decreasing the problem of AMR. In addition, diagnostic and antimicrobial stewardships in the pathology laboratories are useful tools to guide clinicians on patient treatment and to stop the inappropriate use of antibiotics in empirical treatment or narrow antibiotics. Medical Laboratory Scientists are at the forefront of performing antibiotics susceptibility testing in pathology laboratories, thereby helping clinicians to select the appropriate antibiotics for patients suffering from bacterial infections. **Methods:** This cross-sectional study surveyed personal antimicrobial usage, the knowledge and awareness on AMR, and antimicrobial stewardship, as well as barriers to antimicrobial susceptibility testing among medical laboratory scientists in Nigeria using pre-tested and validated questionnaires administered online. The raw data were summarized and exported in Microsoft Excel and further analyzed using IBM SPSS version 26. **Results:** Most of the respondents were males (72%) and 25–35 years old (60%). In addition, the BMLS degree was the highest education qualification most of the respondents (70%) achieved. Of the 59.2% of the respondents involved in antibiotics susceptibility testing, the disc diffusion method was the most commonly used (67.2%), followed by PCR/Genome-based detection (5.2%). Only a small percentage of respondents used the E-test (3.4%). The high cost of testing, inadequate laboratory infrastructure, and a lack of skilled personnel are the major barriers to performing antibiotics susceptibility testing. A higher proportion of a good AMR knowledge level was observed in male respondents (75%) than females (42.9%). The knowledge level was associated with the respondent’s gender (*p* = 0.048), while respondents with a master’s degree were more likely to possess a good knowledge level of AMR (OR: 1.69; 95% CI: 0.33, 8.61). **Conclusion:** The findings of this study indicate that Nigerian medical laboratory scientists had moderate awareness of AMR and antibiotic stewardship. It is necessary to increase investments in laboratory infrastructure and manpower training, as well as set up an antimicrobial stewardship programme to ensure widespread antibiotics susceptibility testing in hospitals, thereby decreasing empirical treatment and the misuse of antibiotics.

## 1. Introduction

Antimicrobial resistance (AMR) is now considered one of the greatest global health threats of the 21st century. Projections show that 10-million people will die from antimicrobial resistance-associated infections globally by 2050 if no intervention strategies are provided [1,2]. A recent Lancet paper [3] estimated that bacterial AMR was associated with 4.95-million deaths and directly responsible for 1.27 million of these deaths in 2019 with *Escherichia coli*, *Staphylococcus aureus*, *Klebsiella pneumoniae*, *Streptococcus pneumoniae*, *Acinetobacter baumannii*, and *Pseudomonas aeruginosa* responsible for 73.4% of the deaths attributable to bacterial AMR deaths. Although bacterial AMR is a global problem, Murray et al. [3] estimated that the highest rates of AMR burden in 2019 were in sub-Saharan Africa. For example, Western sub-Saharan Africa had 27.3 deaths per 100,000, which were regarded as being caused by AMR, as well as 114.8 deaths per 100,000 that were connected to AMR. While the emergence of AMR bacteria is normal when appropriately using antibiotics, many factors accelerate their development [4,5,6]. These factors cause increased selection pressure on bacteria, resulting in more frequent genetic mutations and an increased spread of AMR bacteria and genes [7]. Furthermore, non-adherence to antimicrobial agent prescriptions could increase AMR [8]. In addition, the overuse and misuse of antibiotics in humans and in animal husbandry increases the spread of AMR bacteria and the transfer of resistant genes from animals to humans [8,9,10,11,12,13,14,15,16,17,18,19,20,21]. Poor hygiene practices in humans and animals, as well as poor infection control practices in farms and healthcare settings, increase AMR as they are reservoirs for resistant genes to spread between humans or animals [4,5,22,23].

The appropriate use of antibiotics successfully decreases the morbidity and mortality of infectious diseases [5,24]. However, more Gram-negative and Gram-positive bacteria have developed AMR and, in some cases, have multiple drugs resistance [5]. AMR reduces the efficiency of antibiotic treatment, which causes the ineffectiveness of antimicrobials and increases patients’ morbidity and mortality [25]. Worthy of note is that many different resistant bacteria have been reported in several areas across Nigeria. For instance, a strain of methicillin-resistant *Staphylococcus aureus* has been found in the southwestern part of Nigeria [26]. In addition, an emergence of fluoroquinolone-resistant *Escherichia coli* was reported in Nigeria [2]. Furthermore, two cases of multidrug-resistant tuberculosis were reported in the Southeastern Nigeria [27]. In a different study, Okesola and Oni [28] observed a persistently high bacterial resistance to the commonly used antibiotics in Nigeria and recommended a continued surveillance of changes in antibiotics resistance patterns. Recent evidence indicates a rising trend of antibiotic resistance among common bacterial pathogens in Nigeria [29]. Unfortunately, antimicrobial stewardship programmes (ASP) remain underdeveloped in Nigeria. In addition, there is a high empirical antibiotic use in hospitals and communities, a lack of established antimicrobial stewardship (AMS) teams in most tertiary hospitals, a lack of routine monitoring of antimicrobial susceptibility report and antimicrobial use, easy and unrestricted access to antibiotics, a lack of a framework for nation-wide monitoring of infection prevention and control, and a lack of awareness among healthcare professionals and the general Nigeria public [30,31,32]. In addition, antibiotics are easily accessible in pharmacies, chemists, motor parks, and markets in Nigeria without prescriptions, resulting in the inappropriate use of these antibiotics for prophylactic and therapeutic purposes [33,34,35,36,37].

Antimicrobial stewardship programmes can improve and optimize the use of antibiotics, thereby increasing the cure rates of antibiotic treatment and decreasing the problem of AMR [38]. In addition, performing antibiotic susceptibility testing can help in finding the most effective antimicrobial agent that works on microbial isolates in patients before the treatment, thereby decreasing the risk of AMR [39]. This forms a major component of antimicrobial stewardship and diagnostic stewardship required for a functional AMR surveillance system [40,41,42,43,44] and for the successful implementation of ASP [42,44]. The World Health Organization [40] defined diagnostic stewardship as the “coordinated guidance and interventions to improve the appropriate use of microbiological diagnostics to guide therapeutic decisions. It should promote appropriate, timely diagnostic testing, including specimen collection and pathogen identification, as well as the accurate, timely reporting of results to guide patient treatment”. Efforts geared towards addressing AMR are often targeted at clinicians (who mostly prescribe), nurses, and pharmacists, focusing on optimizing antibiotic prescription to ensure a reduction in overuse and inappropriate usage. However, medical laboratory scientists are often at the forefront of performing antibiotic susceptibility testing, thereby helping clinicians to select the appropriate antibiotics for patients suffering from particular bacterial infections [42]. In addition, the susceptibility and culture data generated by medical laboratory scientists from each hospital could inform the development and implementation of national antimicrobial guidelines [42]. In general, diagnostic and antimicrobial stewardships in the pathology laboratories are useful tools to guide clinicians on patient treatment and to stop the inappropriate use of antibiotics in empirical treatment or narrow antibiotics. Awareness and knowledge regarding AMR and antimicrobial stewardship, as well as understanding the barriers to the ability of medical laboratory scientists to perform antimicrobial susceptibility tests, is critical in preventing the emergence of drug-resistant bacteria, especially in a clinical setting where infection control is of utmost importance. At present, there is a lack of comprehensive information about the awareness and knowledge of AMR and antimicrobial stewardship, as well as the barriers to antimicrobial susceptibility testing among medical laboratory scientists in Nigeria. To expand on the development of interventions to promote responsible antibiotic use and AMS, this study surveyed personal antimicrobial usage, the knowledge and awareness on AMR, and antimicrobial stewardship, as well as barriers to antimicrobial susceptibility testing among medical laboratory scientists in Nigeria.

## 2. Methods

### 2.1. Study Population and Sample Size Calculation

This was a descriptive cross-sectional study of the healthcare professionals registered as medical laboratory scientists who reside and work in Nigeria. These professionals have completed training in medical laboratory science as approved by the Medical Laboratory Science Council of Nigeria (MLSCN) and have at least a Bachelor of Medical Laboratory Science (BMLS) honours degree, an Associate of the Institute of Medical Laboratory Technology (AIMLT) or a Fellow of the Institute of Medical Laboratory Technology (FIMLT). Currently, there are 45 Bachelor of Medical Laboratory Science (BMLS) Training Institutions/ Universities approved by the MLSCN and an estimated 15,000 to 25,000 registered and practicing medical laboratory scientists in Nigeria.

The sample size (*n*) was calculated using the formula as *n* = *Z*^2^*P* (1 − *P*)/*d*^2^ [10], where *n* = sample size, *Z* = *Z* statistic corresponding to a chosen level of confidence, *P* = expected prevalence, and *d* = precision. At a 95% confidence interval, the *Z* statistic was 1.96 and *P* was determined to be 8.3% from a previous national survey of public awareness of antimicrobial resistance in Nigeria [31]. The degree of precision (*d*) was set at 0.05 (in proportion of one) and 5% non-response rate adjusted by considering design effect (1.5). This calculation resulted in a sample size of 117 respondents and was estimated to result in a sufficient number of respondents for determination of the proportions of response to most of the questions.

### 2.2. Questionnaire Design and Administration

The information of the participants was collected using a semi-structured, self-administered questionnaire, which was designed by the authors (Supplementary File S1). We conducted a literature search [27,31,45,46] and developed a 52-question questionnaire, which was validated by randomly sharing it with five medical laboratory scientists in Nigeria. The survey was conducted in the form of an online survey, and the questionnaire was generated by using the Jisc online survey systems, as well as the link of survey. Anyone had access to enter the survey via the survey link shared on the three major Facebook groups used by medical laboratory scientists in Nigeria [Young Medical Laboratory Scientists Forum (YMLSF) youth Wing of AMLSN, Scientists in Development (SIDE), and EBSU Medical Laboratory Science Alumni Network], while only the participants who were over 18 years old, provided informed consent, and confirmed they were medical laboratory scientists who were residing and working in Nigeria at the beginning of the survey in order to continue and complete it effectively.

The questionnaire comprised four-part question format. The first part of the questionnaire collected the demographic characteristics of the medical laboratory scientists, such as gender, age, educational qualification, and employment status. The second part of the questionnaire asked about their working experience and history about the antibiotic susceptibility testing of microbial isolates, including the frequency of antibiotic susceptibility testing, the types of antibiotic susceptibility testing methods, and the barriers of conducting antibiotics susceptibility testing. The questions of the third part collected information about personal use of antibiotics and the attitudes about the use of antibiotics. The final section tested the knowledge of AMR and antimicrobial stewardship by asking “true or false” and “agree or disagree” questions. To assess the participants’ knowledge about antibiotics and AMR, we utilized 24 questions that allowed participants to judge whether the statements were true or false. These statements included the usage of antibiotics on humans and animals, the harm and characteristics of antibiotic resistance, the reason for the development of antibiotic resistance, and the transmission of AMR. In addition, we asked the participants about their general awareness of some key terms, including antibiotic resistance, superbugs, AMR, drug resistance, antibiotic-resistant bacteria, antibiotic stewardship, antimicrobial stewardship, and the Nigeria Centre for Disease Control (NCDC)’s five-point action plan for the responsible use of antimicrobials [47]. Furthermore, the participants were asked about their attitudes towards receiving more information related to AMR and antimicrobial stewardship.

This online survey was conducted from February 2021 to March 2021. The informed consent and agreement by participants were obtained at the beginning of the survey. Respondents participated in the survey voluntarily and had the opportunity to withdraw from the survey at any time.

### 2.3. Ethical Considerations

This study underwent the Coventry University’s ethical approval process, and the Office of Information Security, School of Life Sciences, Coventry University, United Kingdom approved this study (Reference No.: P118890). Informed consent was obtained from the study participants before the commencement of the study and confidentiality ensured.

### 2.4. Data Management and Statistical Analysis

The original data were collected on the Jisc online survey system, and these data were summarized and exported automatically in a Microsoft Excel 2019. The original data were further analyzed using IBM SPSS 26. All numeric or quantitative variables were calculated and expressed as frequencies, proportions, means, and standard deviation. The descriptive statistics of variables were presented by using frequency tables. Binary logistic regression analysis and Chi-square test were used to determine the associations between variables. According to the answer to the question related to the knowledge of AMR and antimicrobial stewardship, a score was created as a dependent variable to determine the knowledge levels of medical laboratory scientists in Nigeria. A total of 24 questions (“agree/disagree” or “true/false”) were used to assess knowledge level, and the score was calculated out by a scoring system. The responses from participants of these 24 questions were scored as “correct” or “incorrect,” and every correct answer from a participant was scored one point; incorrect answer, “I do not know,” or unanswered question scored zero points. The general knowledge score of each respondent was obtained by adding the score gained in each question. Based on this counting method, the knowledge level of participants was categorized into good knowledge if the score was more than 18 points (75%) and poor knowledge if the score equaled 18 points or fewer.

## 3. Results

A total of 50 medical laboratory scientists from the estimated sample size of 117 respondents completed the questionnaire, giving a response rate of 43%. Table 1 shows the demographic characteristics of all respondents. Most of the respondents were male (36/50, 72%). Thirty (60%) respondents were aged between 25 and 34 years, and 40% of respondents were aged between 35 and 44 years. The majority of respondents’ highest educational qualification was a BMLS degree (35/50, 70%), followed by those with a Master’s degree (12/50, 24%). Most of the respondents (28/50, 56%) practiced as a medical laboratory scientist for 5–10 years, whereas more than a quarter of respondents (13/50, 26%) had been registered as a medical laboratory scientist for 10–15 years. Similarly, most of the respondents (21/50, 42%) were employed by a private practice, whereas “Unemployed” and “Government contractual” were each chosen by one participant.

Table 2 displays the experience and history of antibiotic susceptibility testing of respondents. Most of the respondents (37/50, 74%) reported they regularly perform antibiotic susceptibility testing of microbial isolates, but 6% of respondents did not perform this test regularly, and 20% of respondents performed susceptibility testing sometimes. In addition, about 20 out of 50 (40%) respondents only performed antibiotic susceptibility testing on specific samples (6/49, 12.2%) or when medical doctors asked for it (14/49, 28.6%), and 59.2% of respondents always performed antibiotic susceptibility testing of microbial isolates.

Similarly, most of the respondents (38/50, 76%) reported that AMR is a problem in their establishment (Table 2). Furthermore, the majority barrier to widespread antibiotic susceptibility testing in their establishment was the high cost of the test (29/50, 58%) and the inadequacy of the laboratory infrastructure (22/50, 44%). Moreover, 18% of respondents reported that a lack of skilled personnel in their establishment hindered widespread antibiotic susceptibility testing, while two respondents thought it was because of the abuse of antibiotics by patients (Table 2).

Among the responses, only 30% (15/50) of establishments provided formal training in determining the levels of bacterial resistance to antibiotics, while 66% of establishments did not have this training (Table 3). About 50% of respondents could use PCR detection (25/47, 53.2%), the disc-diffusion method (24/47, 51.1%), and MIC and MBC determination (23/47, 48.9%) to detect the AMR. However, this depended on the facilities in their establishment; only 12.8% of respondents could use matrix-assisted laser desorption ionization-time of flight mass spectrometry (MADI-TOF MS), and just 10.6% could use an E-Test. One respondent could perform the Vitek 2 Compact System (Table 3).

More than half of respondents (39/50, 78%) reported that they have used antibiotics in the last year (Table 4). However, only 48% of respondents used antibiotics appropriately by getting a prescription for the antibiotics from a doctor or nurse. Moreover, all respondents thought that they need to take all of the antibiotics as directed by the doctor or pharmacist. Whereas a few respondents (3/49, 6.1%) believed that it is “OK” to use the same antibiotics to help them get better when they contract similar symptoms like before, none of the respondents believed that it is “OK” to use antibiotics from friends or family members, as long as they were used to treat the same illness or similar symptoms (Table 4).

The awareness level of the key terms related to AMR among medical laboratory scientists appeared low. More than half of the respondents did not hear about the NCDC’s five-point action plan for the responsible use of antimicrobials (Table 5). More than a quarter of respondents were unaware of AMR, which is the abbreviation of “antimicrobial resistance.” More than two-thirds of respondents were unaware of superbugs, and less than a third of respondents were aware of “antibiotic stewardship” or “antimicrobial stewardship” (Table 6). Most of the respondents (13/50, 26%) did not know about the definition of “antibiotic stewardship” or “antimicrobial stewardship,” and one-fifth of the respondents (10/50, 20%) had a correct understanding of antibiotic stewardship or antimicrobial stewardship (Table 6).

Most of the respondents appeared to have a desire to further learn about AMR; only a few respondents (2/50, 4%) did not want to receive more information on these issues (Table 6). Most of the respondents wanted to receive more information on “antimicrobial-resistance-detection methods” (35/50, 70%) and “resistance to antibiotics and how resistance develops” (33/50, 66%). Moreover, 44% of respondents wanted to receive more information about guidelines on how to use antibiotics (Table 6).

Most of the respondents chose to get trustworthy information on antibiotics from reliable sources, including “a doctor,” “nurse,” “pharmacy,” “a hospital or other health care facility,” “an official health-related website,” “a TV,” “newspaper/magazine,” “health-related website/blog,” and “radio.” In addition, one respondent reported obtaining information from reputable journals. A few respondents chose to obtain information from unreliable sources, like “family or friends” and “online social network” (Table 7).

All the respondents believed that medical laboratory scientists have a role to play in preventing public health threats posed by antibiotic resistance. Most of the respondents (31/50, 61%) believed that action at all levels is needed to tackle resistance to antibiotics, including at the individual or family level, the organizational level, state level, national level, continental level, and global level. In addition, more than half (73.5%) of respondents believed that antibiotics are widely used in agriculture (Table 8).

Thirty-seven respondents (37/46, 80.4%) correctly associated the overuse of antibiotics with the ineffectiveness of antibiotics (Table 9). Seventeen respondents (17/46, 36.9%) believed that antibiotics are effective in the treatment of cold or flu. In addition, 8.6% of the respondents did not know antibiotics could not kill viruses. More than a quarter of respondents (14/46, 30.4%) thought there is not a risk of getting an antibiotic-resistant infection, as long as antibiotics are taken correctly. Less than half of respondents (19/46, 41.3%) disagreed with the prophylactic antibiotic use on animals, and only half of respondents believed it is correct to decrease the antibiotic use on food-producing animals (Table 9). Over 70% of respondents thought that antibiotic resistance occurs because the body becomes resistant to antibiotics (Table 9). Moreover, all of the respondents believed everyone needs to take responsibility for using antibiotics responsibly and should not keep antibiotics and use them later. All but one of the respondents (45/46, 97.8%) believed that doctors should only prescribe antibiotics when patients need them. Most of the respondents (40/46, 87%) knew that people should use antibiotics only when they are prescribed by a doctor or nurse (Table 9).

In this survey, 30 respondents (65.2%) had good knowledge related to AMR, 16 respondents (34.8%) had poor knowledge, and four respondents did not complete most of the questions in this part (Table 10).

The proportion of respondents with good knowledge of AMR was more in the age group of 35–44 than 25–34 (72.2% vs 60.7%), but the difference between these two age groups was not statistically significant (P = 0.533) (Table 11). Moreover, the results from a PgD respondent and an AMILT or FIMLT respondent were merged into one group as “lower education” with BMLS respondents because the number of these samples was too small for the Chi-square test. The respondents who have a higher education level (Master’s degree) represented a higher proportion (72.7%) of good knowledge of AMR, compared with the respondents (62.9%) who have a lower education (BMLS, AIMLT, FIMLT, PgD). However, this difference was not statistically significant (Table 11). The respondents who had 10 to 15 years of post-qualification experience had a better knowledge level (72.7% vs 62.5%, 63.0%) on AMR compared to other categories, whereas this difference was not statistically significant as *p*= 0.835. The male respondents had a better knowledge level of AMR than the female respondents, and this difference was statistically significant. Thus, the knowledge level of AMR differed significantly between genders (Table 11).

**Figure 1:**
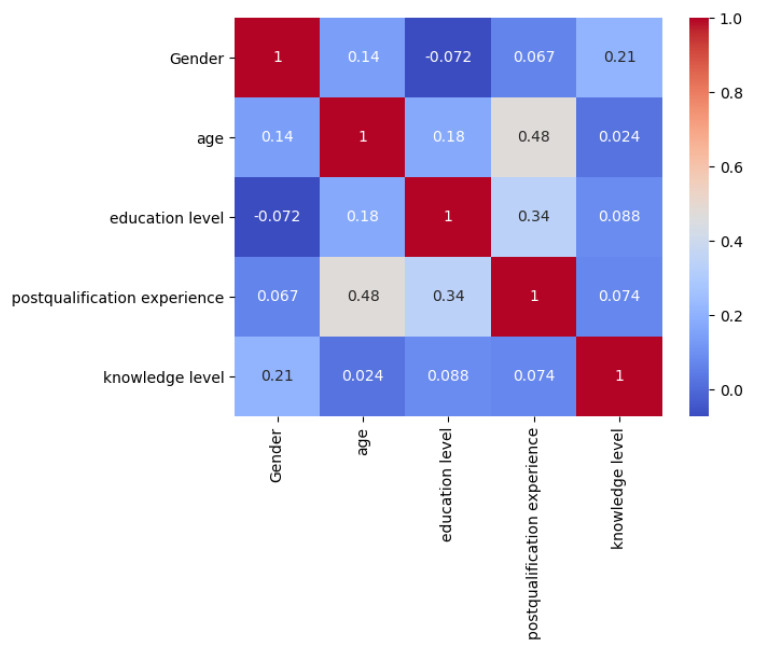
Correlation analysis of the potential factors affecting the knowledge level of AMR and antimicrobial stewardship in respondents. The output shows the correlation coefficients between all pairs of variables. The values range from −1 to 1, where −1 indicates a perfect negative correlation, 0 indicates no correlation, and 1 indicates a perfect positive correlation.

The binary logistic regression analysis (Table 12) revealed the potential factors that affect the knowledge level of AMR and antimicrobial stewardship among medical laboratory scientists in Nigeria. Male respondents (OR: 2.81; 95% CI: 0.73, 10.86) were 2.81 times more likely to possess a good knowledge level of AMR and antimicrobial stewardship than female respondents. However, the knowledge status was not significantly associated with gender as the *p* value is higher than 0.05 (*p* = 0.134). In addition, the respondents who had a Master’s degree (OR: 1.69; 95%CI: 0.33, 8.61) were more likely to possess a good knowledge level of AMR than other categories. In addition, the respondents who had 10–15 years post-qualification experience were more likely to have a better knowledge status (OR: 1.47, 95%CI: 0.14, 15.98), whereas the knowledge level of AMR and antimicrobial stewardship were not significantly affected by a respondent’s post-qualification experience (*p* = 0.882, *p* = 0.751), education level (*p* = 0.527), and age (*p* = 0.697), as their *p* value was higher than 0.05. We also performed correlation analysis to calculate the correlation coefficient between pairs of variables to investigate the strength and direction of their association (Figure 1). Weak correlation was observed across the majority of the features, with post-qualification experience and age having the strongest correlation in the dataset.

## 4. Discussion

The emergence of antibiotic resistance in bacteria is becoming more frequent; therefore, AMR is attracting more attention worldwide. The rising trend of antimicrobial resistance has become a global threat to human and animal health, as well as modern medicine [1]. It is estimated that in 2019, more than 1.2-million individuals died globally as a result of infections caused by bacteria resistant to antibiotics, which is more than the annual death toll associated with the combination of malaria or AIDS [3]. A correct and timely use of antibiotics can effectively kill bacteria and prevent and fight infection. However, the abuse and overuse of antimicrobial agents make bacteria faster in developing resistance to antimicrobial agents. The irrational use of antimicrobial agents has led to the gradual loss of bacteria’s sensitivity to antimicrobial agents and the spread of AMR, thereby significantly affecting healthcare and economies worldwide [1]. Against this background, this study examined personal antimicrobial usage, the knowledge and awareness on AMR and antimicrobial stewardship, and the barriers to antimicrobial susceptibility testing among medical laboratory scientists in Nigeria. To the best of authors’ knowledge, this is the first study to access the knowledge of AMR and antimicrobial stewardship, as well as the barriers to antimicrobial susceptibility testing among medical laboratory scientists in Nigeria.

In this study, a total of 50 medical laboratory scientists completed the questionnaire out of the estimated sample size of 117 participants, giving a response rate of 43%. In a meta-analysis of the response rates of online surveys in published research, Wu et al. [48] examined 1071 independent online survey response rates and reported that the average online survey response rate is 44.1%. In addition, Wu et al. [48] demonstrated that sending an online survey to more participants did not generate a higher response rate. Previous meta-analyses had average response rates of online surveys of 48.3% [49], 34% [50], 39.6% [51], 34.2% [52], and 42.8% [53]. Previous research also showed that online surveys produce an 11%–12% lower response rate than other types of surveys [54,55,56]. However, it has been demonstrated that lower response rates are not associated with an increase in the non-response error [57,58]. Interestingly, Fosnacht et al. [59] reported that surveys with a smaller sample size (i.e., less than 500) need approximately 20%–25% response rates to generate confident estimates and remain reliable. Therefore, the information generated from this research will be considered relevant.

Most of the laboratory scientists in this study were between 25 and 44 years old, which probably explains why more than 70% of the respondents have been registered as medical laboratory scientists within 10 years. Most of the respondents were employees of private practice or government (78%), while a small number of respondents were higher education teachers (8%) and non-governmental organization employees (12%). This distribution of employment status was similar to a study conducted in Nigeria by Adekanye et al. [60]. However, 65% of the respondents were unfamiliar with the terms of antibiotic stewardship and antimicrobial stewardship, compared to 37% reported in a similar study in Nigeria [60]. At present, there is no explanation about the exact reason for this difference. However, the sample size of the current study was smaller than the study by Adekanye et al. [60], which targeted participants across Nigeria. Thus, the current study can be considered less representative of the general population. Nevertheless, our study highlighted that the medical laboratory scientists in Nigeria still have insufficient awareness of the concept of antimicrobial stewardship or antibacterial stewardship.

More than 20% of respondents reported that they do not regularly perform antibiotics susceptibility testing, and less than 60% of respondents were always performing antibiotics susceptibility testing. In addition, most of the respondents were still using the disc-diffusion method and dilution or broth method for antibiotic susceptibility testing. Most of the respondents reported that the high cost of testing, inadequate laboratory infrastructure, and a lack of skilled personnel are the main barriers to performing antibiotic susceptibility testing, while two respondents reported the abuse of antibiotics in patients was a barrier as well because patients use antibiotics before testing. It has been demonstrated that the main challenges hindering the implementation of antimicrobial stewardship programmes in low-middle income countries were a lack of access to laboratory diagnosis, quality antimicrobials, trained and competent healthcare professionals, knowledge among practitioners, and healthcare facility infrastructure [42]. It has been postulated that one of the major drivers of AMR is the inadequate pathology laboratory infrastructure, resulting in poor microbiological and antimicrobial susceptibility testing [3]. Coincidentally, 76% of respondents reported AMR as a problem in their establishment. These findings suggest that there should be increased investment in laboratory infrastructure in order to perform faster and more accurate antibiotic susceptibility testing, such as PCR- based detection, thereby decreasing the cost per test, which may be helpful in antibiotic susceptibility testing being conducted more widely. In addition, an improvement in the education and training of medical laboratory practitioners would help to increase the number of medical practitioners with the necessary skills and knowledge as well. Therefore, these two strategies may be helpful in promoting the responsible use of antibiotics by widely performing antibiotic susceptibility testing before the use of antibiotics of patients and impede the appearance of AMR [60]. In previous studies in low-resource settings, healthcare professionals have recommended the provision of protocols and guidelines, more AMR training courses, improved laboratory and diagnostic services, and a greater availability of patient educational materials in order to support the long-term delivery and sustenance of antibiotic stewardship programmes [61,62,63,64,65]. Obviously, improving the knowledge of the appropriate antibiotic use of the public may help to decrease the self-use of antibiotics before the antibiotic susceptibility testing, which is important to decrease the abuse of antibiotics.

Seventy-eight percent of respondents reported they have used antibiotics in the last year, but only 48% of respondents claimed that they received this antibiotic from a doctor’s or nurse’s prescription. Comparing with the result from a similar study that surveyed veterinary students in Nigeria [66], a similar proportion (75.6%) of respondents had used antibiotics in the last year, while a higher proportion (60.7%) of respondents received antibiotics from a doctor’s prescription. However, whether this difference between veterinary students and medical laboratory scientists is related to the training they received has not been explored. Interestingly, 40 (87%) respondents knew that they should use antibiotics only when they are prescribed by a doctor or nurse, but only 24 (48%) respondents followed that rule. Based on these findings in the current study, there is a need for an improvement of the understanding of the correct antibiotic use among medical laboratory scientists in order to decrease the abuse of antibiotics.

All but one of the respondents claimed they know about “antimicrobial resistance,” while more than 70% of respondents believed that antibiotic resistance occurs when your body becomes resistant to antibiotics and they no longer work as well. In addition, more than 30% of respondents did not hear about “AMR,” and these were respondents who either did not perform antimicrobial susceptibility test or who only performed susceptibility testing sometimes. In addition, a low proportion of respondents knew about the key terms “superbugs” (30.6%), “NCDC five-point action plan for responsible use of antimicrobials” (38%), and either “antibiotic stewardship” or “antimicrobial stewardship” (32%). Previous studies from Odetokun et al. [66] and Anyanwu et al. [65] reported a similar result. Additionally, another previous survey targeted on human and animal health students in the United Kingdom also reported a low proportion of respondents who had heard of either “antibiotic stewardship” or “antimicrobial stewardship” [67]. The low level of understanding of AMR may be attributed to the lack of in-depth AMR education for medical laboratory scientists and the lack of courses in medical laboratory science [66]. Similarly, a study targeted at medical students in France pointed out a need for more and deeper education on the concept of AMR [68]. Furthermore, a previous study has pointed out that the awareness of the NCDC five-point action plan may be related to the appropriate use of antibiotics [60].

Most of the respondents (91.4%) knew that antibiotics cannot kill viruses, but more than 30% of respondents believed that antibiotics are effective in the treatment of the cold or flu. Similar results were reported from a previous study in Nigeria by Alex [46] and Adekanye et al. [60]. These findings indicated that there were some deficiencies in the training and teaching on the course of medical laboratory science, especially the education of microbiology and antimicrobial resistance [46,60]. Moreover, most people knew that the inappropriate use of antibiotics in animals can result in a negative impact on human health. However, only 56.5% of people agreed farmers should deliver fewer antibiotics to food-producing animals, and 41.3% of respondents thought prophylactic antibiotics are an appropriate alternative to protect animal health. In addition, a small proportion of respondents (17.4%) thought a withdrawal period does not have to be observed for milking cows treated with antibiotics, such as penicillin, before milk can be consumed. However, a missing withdrawal period after antibiotic treatment may result in antibiotic residues in the animal product, which will be ingested by humans, thereby causing potential risks to human health [69]. For example, this can cause antibiotic-related allergies and hypersensitivity reactions, as well as the development of AMR [60,70,71]. Additionally, only 30% of establishments provided formal training in determining the levels of bacterial resistance. The current study did not explore the association between additional training and appropriate antibiotics use. However, a relationship between having additional education or training on AMR and appropriate antibiotic use has been demonstrated by Adekanye et al. [60]. This finding may explain why most of the respondents did not use antibiotics appropriately [60].

In the current study, 34.8% of respondents had a poor AMR knowledge level. It was observed that male respondents have a better knowledge level of AMR than female respondents as they have a higher proportion of respondents (75% vs 45.9%) with a good knowledge status. In addition, those who had a higher education level, belong to an older age group, and had longer post-qualification experience showed a higher AMR knowledge status. However, according to the result of the Chi-square and binary logistic regression analysis, only a significant difference of the knowledge status was observed between respondents’ gender in the Chi-square test (*p* = 0.048), while the knowledge status was not associated with gender when tested by binary logistic regression analysis (*p* = 0.134). Moreover, there was no significant difference and association between age, education level, post-qualification experience, and AMR knowledge status. Another study has also reported a better AMR knowledge level in male respondents (*p* = 0.035) [46]. However, a correlation analysis showed that post-qualification experience and age had the strongest correlation in the dataset. This finding in the current study may be caused by the small sample size. Moreover, most of the respondents (96%) represented an enthusiastic attitude for further learning related to AMR, and most of the respondents (88%) chose to get information on AMR from reliable sources. This finding may help to increase the awareness of AMR and AMS among medical laboratory scientists in the future. 

## 5. Conclusions

In conclusion, the surveyed Nigerian medical laboratory scientists had a moderate awareness of AMR and antibiotic stewardship but a willingness to improve. In addition, the respondents demonstrated a positive attitude towards AMR control. Respondents believed that they could help to prevent the public health threats posed by AMR as medical laboratory scientists. The lack of skilled personnel and funding hindered the conduct of antibiotic susceptibility testing, which will negatively impact antibiotic prescription patterns in associated local hospitals and subsequently affect AMR and AMS in Nigeria. Furthermore, it was determined that there is a need for deeper studying and training on microbiology, pharmacology, and medicine for medical laboratory scientists in order to increase the awareness of AMR. Overall, the information from this research will be useful in the design and implementation of collaborative ASPs within Nigerian hospitals, as well as inform the development of training and diagnostic stewardship improvement strategies and other policies geared towards the reduction of antibiotic resistance in Nigeria. However, this study has some limitations, including a small sample size as well as a narrow recruitment scope because the respondents were only recruited from Facebook groups.

## Figures and Tables

**Table 1:** Demographics of respondents.

Demographic Characteristics	Response	Frequency (*N* = 50)	Percentage (%)
Gender	Female	14	28
	Male	36	72
Age group	18–24 years old	0	0
	25–34 years old	30	60
	35–44 years old	20	40
	45–54 years old	0	0
	55–65 years old	0	0
	More than 65 years old	0	0
Highest level of education	AIMLT or FIMLT	1	2
	BMLS	35	70
	Other Bachelor’s degree	0	0
	Postgraduate Diploma	1	2
	Master’s degree	12	24
	Doctorate degree	1	2
	Other	0	0
Years registered as a medical laboratory scientist	0–5 years	9	18
	6–10 years	28	56
	11–15 years	13	26
	16–20 years	0	0
	21 and above	0	0
	Prefer not to say	0	0
Employment status	Private practice	21	42
	NGO employee	6	12
	Government employee	17	34
	Research	0	0
	Teaching	4	8
	Unemployed	1	2
	Government contractual	1	2

**Table 2:** The experience and history of antibiotics susceptibility testing of respondents.

Question	Response	Frequency	Percentage (%)
Do you regularly perform antibiotics susceptibility testing of microbial isolates? (*N* = 50)	Yes	37	74
	No	3	6
	Sometimes	10	20
When do you perform antibiotics susceptibility testing of microbial isolates? (*N* = 49)	It is always performed.	29	59.2
	It is performed on specific samples.	14	28.6
	It is performed only when medical doctors ask for it.	6	12.2
	Not performed at all.	0	0
	Other.	0	0
Is antimicrobial resistance a problem in your establishment?	Yes	38	76
	No	10	20
	Don’t know	2	4
What are barriers to widespread antibiotics susceptibility testing in your establishment?	High cost of tests	29	58
	Lack of skilled personnel	9	18
	Inadequate laboratory infrastructure	22	44
	Limited access to rapid/point-of-care diagnostic tests	9	18
	Don’t know	3	6
	Other	4	8

**Table 3:** The situations of establishment of respondents.

Question	Response	Frequency	Percentage (%) *
Is formal training in determining the levels of bacterial resistance to antibiotics available in your establishment? (*N* = 50)	Yes	15	30
	No	33	66
	Don’t know	2	4
Considering the facilities available in your establishment, which antimicrobial resistance detection method would be most relevant for you to learn? (*N* = 47)	PCR/Genome-based detection	25	53.2
	Disc diffusion method	24	51.1
	MIC and MBC determination (Dilution or broth method)	23	48.9
	Matrix-assisted laser desorption ionization–time of flight mass spectrometry (MALDI-TOF MS)	6	12.8
	E-Test	5	10.6
	Other	1	2.1

* 100% would represent that all this question’s respondents chose that option.

**Table 4:** Personal use of antibiotics among medical laboratory scientists in Nigeria.

Parameters	Response	Frequency	Percentage (%)
Last antibiotic use (*N* = 50)	Last month	22	44
	6 months ago	10	20
	In the last year	7	14
	More than a year ago	7	14
	Can’t remember	4	8
	Never	0	0
Did you get a prescription for the antibiotics from a doctor or nurse (*N* = 50)	Yes	24	48
	No	26	52
	Can’t remember	0	0
	When you feel better	0	0
When do you think you should stop taking antibiotics once you have begun treatment? (*N* = 49)	When you have taken all of the antibiotics as directed by the Doctor or pharmacist	49	100
	Don’t know	0	0
	Other	0	0
It’s okay to buy the same antibiotics, or request these from a doctor, if you’re sick and they helped you get better when you had the same symptoms before (*N* = 49)	True	3	6.1
	False	46	93.9
	Don’t know	0	0

**Table 5:** Awareness level among medical laboratory scientists on key terms relating to AMR.

Key Terms	No (%)	Yes (%)
Nigeria Centre for Disease Control (NCDC) 5-point action plan for responsible use of antimicrobials	31 (62)	19 (38)
Antibiotic resistance	2 (4.1)	47 (95.39)
Superbugs	34 (69.4)	15 (30.6)
Antimicrobial resistance	1 (2.1)	48 (97.9)
AMR	18 (36.7)	31 (63.3)
Drug resistance	2 (4.1)	47 (95.9)
Antibiotic-resistance bacteria	3 (6.1)	46 (93.9)
Antibiotic stewardship/ antimicrobial stewardship	34 (65)	16 (32)

**Table 6:** Understanding levels of antibiotic stewardship and information respondents want to receive more.

Question and Type of Information	Response	Frequency	Percentage * (%)
Which of the following do you think best defines antibiotic stewardship or antimicrobial stewardship? (*N* = 50)	A systematic effort to educate and persuade prescribers of antibiotics to follow evidence-based prescribing, in order to stem antibiotic overuse, and thus antibiotic resistance.	2	4
	A coordinated intervention designed to improve and measure the appropriate use of antibiotics by promoting the selection of the optimal antimicrobial drug regimen, dose, duration of therapy and route of administration.	2	4
	Optimal selection, dosage, and duration of antibiotic treatment that results in the best clinical outcome for the treatment or prevention of infection, with minimal toxicity to the patient and minimal impact on subsequent resistance	6	12
	All of the above	27	54
	None of the above	0	0
	Don’t know	13	26
Which of the following topics would you like to receive more information on? Resistance to antibiotics		26	52
Guidelines on how to use antibiotics		22	44
Medical conditions for which antibiotics are used		18	36
Prescription of antibiotics		26	52
Critically important antimicrobials		26	52
Resistance to antibiotics and how resistance develops		33	66
Antimicrobial resistance detection methods		35	70
Links between the health of humans, animals and the environment		27	54
Don’t want to receive more information on these issues		2	4
Don’t know		0	0

* 100% would represent that all this question’s respondents chose that option.

**Table 7:** The sources of information that respondents trusted to get information on antibiotics.

Sources of Information	Frequency	Percentage (%) *
A doctor	33	66
A nurse	13	26
A pharmacy	38	76
A hospital or other health care facility	19	38
Family or friends	1	2
An official health-related website (e.g., a website set up by the national government/public health body)	31	62
A health-related website/blog	15	30
Online social network	5	10
TV	1	2
Newspapers/magazines	3	6
Radio	2	4
Not interested in finding information on antibiotics	0	0
Other	1	2

* 100% would represent that all this question’s respondents chose that option.

**Table 8:** Attitude of respondents on AMR and antimicrobial stewardship.

Question	Response	Frequency	Percentage (%) *
Do you believe as a Medical Laboratory Scientist, you have a role to play in preventing public health threats posed by antibiotic resistance? (*N* = 50)	Yes	50	100
	No	0	0
At what level do you believe it is most effective to tackle resistance to antibiotics? (*N* = 50)	At Individual level or within family	17	34
	At the organisational level	0	0
	At state level	0	0
	At national level	0	0
	At continental level	0	0
	At global level	2	4
	Action at all levels is needed Don’t know	31 0	62 0
	Yes	36	73.5
Do you think antibiotics are widely used in agriculture (including in food-producing animals) in your country? (*N* = 49)	No	9	18.4
	Don’t know	4	8.2

* 100% would represent that all this question’s respondents chose that option.

**Table 9:** Knowledge of AMR and antimicrobial stewardship among the respondents.

Variable	Response	Frequency	Percentage (%)
Antibiotic-resistant infections could make medical procedures like surgery, organ transplants and cancer treatment much more dangerous	True	44	95.7
	False	1	2.2
	I don’t know	1	2.2
Bacteria which are resistant to antibiotics can be spread from one person to the other	True	42	91.3
	False	4	8.7
	I don’t know	0	0
Antibiotic resistance is only a problem for people who take antibiotics regularly	True	8	17.4
	False	38	82.6
	I don’t know	0	0
Antibiotic resistance is an issue that affect other countries, but not here	True	2	4.3
	False	44	95.7
	I don’t know	0	0
Antibiotic resistance is an issue that could affect me or my family	True	43	93.5
	False	3	6.5
	I don’t know	0	0
If bacteria are resistant to antibiotics, it can be very difficult or impossible to treat the infections they cause	True	45	97.8
	False	1	2.2
	I don’t know	0	0
Infections caused by antibiotics resistant bacteria can be very difficult to treat	True	43	95.6
	False	2	4.4
	I don’t know	0	0
Many infections are becoming increasingly resistant to treatment by antibiotics	True	43	95.6
	False	1	2.2
	I don’t know	1	2.2
Antibiotic resistance occurs when your body becomes resistant to antibiotics and they no longer work as well	True	33	70.2
	False	14	29.8
	I don’t know	0	0
Healthy people and animals can carry antibiotic resistant bacteria	Agree	40	86.9
	Disagree	5	10.9
	I don’t know	1	2.2
Prophylactic antibiotics are an appropriate alternative to protect animal health	Agree	19	41.3
	Disagree	19	41.3
	I don’t know	8	17.4
Farmers should give fewer antibiotics to food-producing animals	Agree	26	56.5
	Disagree	11	23.9
	I don’t know	9	19.6
Inappropriate use of antibiotics in animals can result in negative impact on human health	Agree	39	84.8
	Disagree	5	10.9
	I don’t know	2	4.3
People should use antibiotics only when they are prescribed by a doctor or nurse	Agree	40	87
	Disagree	6	13
	I don’t know	0	0
People should not keep antibiotics and use them later for other illnesses	Agree	46	100
	Disagree	0	0
	I don’t know	0	0
Doctors should only prescribe antibiotics when they are needed	Agree	45	97.8
	Disagree	1	2.2
	I don’t know	0	0
Everyone needs to take responsibility for using antibiotics responsibly	Agree	46	100
	Disagree	0	0
	I don’t know	0	0
I am not at risk of getting an antibiotic resistant infection, as long as I take my antibiotics correctly.	Agree	31	67.4
	Disagree	14	30.4
	I don’t know	1	2.2
Antibiotics kill viruses	Agree	2	4.3
	Disagree	41	91.4
	I don’t know	2	4.3
Antibiotics are effective in the treatment of cold or flu	Agree	14	30.4
	Disagree	29	63
	I don’t know	3	6.5
Overuse of antibiotics makes them become ineffective	Agree	37	80.4
	Disagree	7	15.2
	I don’t know	2	4.3
A withdrawal period has to be strictly observed in treated poultry before any poultry product is passed as fit for human consumption	Agree	41	89.1
	Disagree	1	2.2
	I don’t know	4	8.7
A withdrawal period does not have to be observed for milking cows treated with antibiotics such as penicillin before milk can be consumed	Agree	8	17.4
	Disagree	31	67.4
	I don’t know	7	15.2
Pharmaceutical companies should develop new antibiotics	Agree	38	80.9
	Disagree	7	14.9
	I don’t know	2	4.3

**Table 10:** The knowledge status of AMR and antimicrobial stewardship of respondents.

Knowledge Status	Frequency (*N* = 46)	Percentage (%)
Good knowledge	30	65.2
Poor Knowledge	16	34.8

**Table 11:** Possible factors of respondent associated with knowledge status of AMR and antimicrobial stewardship in respondents.

Variable	Knowledge Status Good Poor	Chis Square (χ2)	*p*-Value
Age		0.640	0.533
25–34	17 (60.7%) 11 (39.3%)		
35–44	13 (72.2%) 5 (27.8%)		
Gender		4.436	0.048 *
Male	24 (75%) 8 (25%)		
Female	6 (42.9%) 8 (57.1%)		
Education level		0.359	0.722
Master’s degree	8 (72.7%) 3 (27.3%)		
Lower education Level (including BMLS, AIMLT or FIMLT, and PgD)	22 (62.9%) 13 (37.9%)		
Post-qualification experience		0.360	0.835
0–5 years	5 (62.5%) 3 (37.5%)		
6–10 years	17 (63.0%) 10 (37.0%)		
11–15 years	8 (72.7%) 3 (27.3%)		

* Statistically significant.

**Table 12:** The binary logistic regression analysis of the potential factors affects the knowledge level of AMR and antimicrobial stewardship in respondents.

Variable	Knowledge Status Good (%) Poor (%)	OR	95% CI	*p*-Value
Age				
25–34	17 (60.7) 11 (39.3)	1.00		
35–44	13 (72.2) 5 (27.8)	0.74	0.16–3.41	0.697
Gender				
Male	24 (75) 8 (25)	2.81		
Female	6 (42.9) 8 (57.1)	1.00	0.73–10.86	0.134
Education level				
Lower education (including BMLS, AIMLT or FIMLT, and PgD)	22 (62.9) 13 (37.9)	1.00		
Master’s degree	8 (72.7) 3 (27.3)	1.69	0.33–8.61	0.527
Post-qualification experience				
0–5 years	5 (62.5) 3 (37.5)	1.00	0.16–4.97	0.882
6–10 years	17 (63.0) 10 (37.0)	0.88	0.14–15.98	0.751
11–15 years	8 (72.7) 3 (27.3)	1.47		

## Data Availability

All data generated or analyzed during this study are included in this published article [Supplementary File S1]. Original data are available from the corresponding author upon reasonable request.

## Supplementary Materials

The following supporting information can be downloaded at: https://www.mdpi.com/article/10.3390/antibiotics12050815/s1, Supplementary File S1: Survey questionnaire.

## Abbreviations

WHO: World health organization; AMR: Antimicrobial resistance; AMS: Antimicrobial stewardship; ASP: Antimicrobial stewardship programme; SPSS: Statistical package for social sciences; NCDC: Nigeria Centre for Disease Control; YMLSF: Young Medical Laboratory Scientists Forum; SIDE: Scientists in Development; EBSU: Ebonyi State University; MLSCN: Medical Laboratory Science Council of Nigeria; BMLS: Bachelor of Medical Laboratory Science; AIMLT: Associate of the Institute of Medical Laboratory Technology; FIMLT: Fellow of the Institute of Medical Laboratory Technology; OR: Odds ratio; CI: Confidence interval; NGO: Non-Governmental Organization; PgD: Postgraduate Diploma.
