# Supplementary material for: Awareness and Knowledge of Antimicrobial Resistance, Antimicrobial Stewardship and Barriers to Implementing Antimicrobial Susceptibility Testing among Medical Laboratory Scientists in Nigeria: A Cross-Sectional Study
Source: Antibiotics (Basel). 2023 Apr 26;12(5):815. doi: 10.3390/antibiotics12050815 (PMC10215829; doi:10.3390/antibiotics12050815)
Supplement: Supplementary file 1 [file antibiotics-12-00815-s001.zip › antibiotics-2358686-supplementary.pdf]

Supplementary File 1: Survey Questionnaire

A survey of awareness, knowledge and practices on antibiotics use, antimicrobial resistance, antimicrobial stewardship and barriers on antimicrobial susceptibility testing among Medical Laboratory Scientists in Nigeria.

The aim of this study is to **investigate the awareness, Knowledge and Attitudes towards antimicrobial resistance and antimicrobial stewardship and the barriers to antimicrobial susceptibility testing among Medical Laboratory Scientists in Nigeria**. The study is being conducted by **Mr. Sheng Huang and Dr Ukpai Eze at Coventry University**. Your participation in the survey is entirely voluntary, and you can opt out at any stage by closing and exiting the browser. If you are happy to take part, please answer the following questions relating to the antimicrobial resistance and antimicrobial stewardship. Your answers will help us to assess the awareness of antimicrobial resistance/stewardship and the factors hindering susceptibility testing practices among Medical Laboratory Scientists in Nigeria. The survey should take approximately 10-15 minutes to complete.

Your answers will be treated confidentially and the information you provide will be kept anonymous in any research outputs/publications. Your data will be held securely on the lead researcher's university encrypted OneDrive account. The project has been reviewed and approved through the formal Research Ethics procedure at Coventry University (protocol code: P118890 and date of approval: 15/02/2021). For further information, or if you have any queries, please contact the investigator **Sheng Huang** at Huangs24@uni.coventry.ac.uk or the project supervisor **Dr Ukpai Eze** at ad2179@coventry.ac.uk. Thank you for taking the time to participate in this survey. Your help is very much appreciated.

Please select **YES** or **NO** to confirm your agreement with the statement below "I have read and understood the above information. I understand that, because my answers will be fully anonymised, it will not be possible to withdraw them from the study once I have completed the survey. I agree to take part in this questionnaire survey and give my consent. I confirm that I am aged 18 or over. "

**Yes*

**No*

As this survey is designed to investigate the awareness, knowledge and practices of antimicrobial resistance/stewardship and barriers to antimicrobial susceptibility testing among Medical Laboratory Scientists in Nigeria, so the participants need to be Medical Laboratory Scientists living in Nigeria.

So, I will ask:

1.Are you a Medical Laboratory Scientist living in Nigeria?

- Yes
- No

If you answered YES to question 1, then proceed and answer the following questions below.

2. What is your gender?

• Male
• Female
• Prefer not to say
• Others, please specify ___________________________

3. Please indicate your age group: Tick as appropriate

• 18-24 years old

• 25-34 years old
• 35-44 years old
• 45-54 years old
• 55-64 years old
• 65 years or older
• Prefer not to say

4. What is the highest educational qualification you attained?

• AIMLT or FIMLT

• Bachelor of Medical Laboratory Science (B.MLS)
• Other Bachelor's degree
• Postgraduate Diploma
• Master's degree
• Doctorate degree
• Others, please specify ___________________________

5. For how many years have you been registered as a Medical Laboratory Scientist? Tick as appropriate.

• 0-<5 years
• 5-10 years
• 10-15 years
• 15-20 years
• 21 and above
• Prefer not to say

6. Which of the following best describes your employment status? Tick as appropriate.
• Private practice
• Non-governmental organisation employee
• Government employee
• Research
• Teaching
• Other, please specify ____

7. Do you regularly perform antibiotics susceptibility testing of microbial isolates?
• Yes
• No
• Sometimes

8. When do you perform antibiotics susceptibility testing of microbial isolates?
• It is always performed
• It is performed on specific samples
• It is performed only when medical doctors ask for it
• It is not performed at all
• Other responses (Pls, specify) ---------------------------

9. What tests do you currently use to diagnose antimicrobial resistance? (Select all that apply)
• Disc diffusion method
• MIC and MBC determination (Dilution or broth method)
• E-Test
• PCR/Genome-based detection
• Other tests (Please, specify)

10. Is antimicrobial resistance a problem in your establishment?
• Yes
• No
• Don't know

11. What are barriers to widespread antibiotics susceptibility testing in your establishment? (Select all that applies)
• High cost of tests
• Lack of skilled personnel
• Inadequate laboratory infrastructure
• Limited access to rapid/point-of-care diagnostic tests
• Don't know
• Other reasons (Pls, specify) ---------------------------------

12. Is a formal training in determining the levels of bacterial resistance to antibiotics available in your establishment?

• Yes
• No
• Don’t know

13. Considering the facilities available in your establishment, which antimicrobial resistance detection method would be most relevant for you to learn? (Select all that applies)

• Disc diffusion method
• MIC and MBC determination (Dilution or broth method)
• E-Test
• PCR/Genome-based detection
• Matrix-assisted laser desorption ionization–time of flight mass spectrometry (MALDI-TOF MS)
• Other tests (Please, specify)

14. Have you read or heard of the Nigeria Centre for Disease Control (NCDC) 5-point action plan for responsible use of antimicrobials?

• Yes
• No

15. When did you last take antibiotics?

• In the last month
• In the last 6 months
• In the last year
• More than a year ago
• Never
• Can’t remember

16. On any occasion that you have taken antibiotics, did you get a prescription for the antibiotics from a doctor or nurse?

• Yes
• No
• Can’t remember

17. When do you think you should you stop taking antibiotics once you have begun a course of treatment?

• When you feel better
• When you’ve taken all of the antibiotics as directed by the Doctor or pharmacist
• Don’t know
• Others, please specify ………………………………………..

18. Do you think this statement is 'true' or 'false'?
"It's okay to use antibiotics that were given to a friend or family member, as long as they were used to treat the same illness"
- True
- False
- Don't know

19. Do you think this statement is 'true' or 'false'?
"It's okay to buy the same antibiotics, or request these from a doctor, if you're sick and they helped you get better when you had the same symptoms before"
- True
- False
- Don't know

20. Have you heard of any of the following terms? ***Select all that apply***.

- Antibiotic resistance
- Superbugs
- Antimicrobial resistance
- AMR
- Drug resistance
- Antibiotic-resistant bacteria
- Don't know

21. Have you ever heard of the term 'antibiotic stewardship' or 'antimicrobial stewardship'?
- Yes
- No
- Don't know

22. Which of the following do you think best defines antibiotic stewardship or antimicrobial stewardship?
- A systematic effort to educate and persuade prescribers of antibiotics to follow evidence-based prescribing, in order to stem antibiotic overuse, and thus antibiotic resistance.
- A coordinated intervention designed to improve and measure the appropriate use of antibiotics by promoting the selection of the optimal antimicrobial drug regimen, dose, duration of therapy and route of administration.
- Optimal selection, dosage, and duration of antibiotic treatment that results in the best clinical outcome for the treatment or prevention of infection, with minimal toxicity to the patient and minimal impact on subsequent resistance
- All of the above
- None of the above
- Don't know

23. Do you think antibiotics are widely used in agriculture (including in food-producing animals) in your country?

• Yes
• No
• Don’t know

24. Of the following, which topics would you like to receive more information on? Select all that apply.

• Resistance to antibiotics
• Guidelines on how to use antibiotics
• Medical conditions for which antibiotics are used
• Prescription of antibiotics
• Critically important antimicrobials
• Resistance to antibiotics and how resistance develops
• Antimicrobial resistance detection methods
• Links between the health of humans, animals and the environment
• Don’t want to receive more information on these issues
• Don't know

25. Which of the following sources of information would you use in order to get trustworthy information on antibiotics (*Select all that apply*)

• A doctor
• A nurse
• A pharmacy
• A hospital or other health care facility
• Family or friends
• An official health-related website (e.g. a website set up by the national government/ public health body)
• A health-related website/blog
• Online social network
• TV
• Newspapers/magazines
• Radio
• Not interested in finding information on antibiotics
• Other source of information (please specify)……………….

26. At what level do you believe it is most effective to tackle resistance to antibiotics?

• At Individual level or within family
• At the organisational level
• At state level
• At national level
• At continental level
• At global level
• Action at all levels is needed

• Don't know

27. What do you think about the following statements? (*Please, answer all the questions*)

	Yes	No	I don’t know
Antibiotic-resistant infections could make medical procedures like surgery, organ transplants and cancer treatment much more dangerous			
Bacteria which are resistant to antibiotics can be spread from one person to the other			
Antibiotic resistance is only a problem for people who take antibiotics regularly			
Antibiotic resistance is an issue that affect other countries, but not here			
Antibiotic resistance is an issue that could affect me or my family			
If bacteria are resistant to antibiotics, it can be very difficult or impossible to treat the infections they cause			
Infections caused by antibiotics resistant bacteria can be very difficult to treat			
Many infections are becoming increasingly resistant to treatment by antibiotics			
Antibiotic resistance occurs when your body becomes resistant to antibiotics and they no longer work as well			

28. Please, answer the following questions? (*Answer all the questions by selecting the appropriate scale*)

	Yes	No	I don’t know
Healthy people and animals can carry antibiotic resistant bacteria			
Prophylactic antibiotics are an appropriate alternative to protect animal health			
Farmers should give fewer antibiotics to food-producing animals			
Inappropriate use of antibiotics in animals can result in negative impact on human health			
People should use antibiotics only when they are prescribed by a doctor or nurse			
People should not keep antibiotics and use them later for other illnesses			

Doctors should only prescribe antibiotics when they are needed			
Everyone needs to take responsibility for using antibiotics responsibly			
I am not at risk of getting an antibiotic resistant infection, as long as I take my antibiotics correctly.			
Antibiotics kill viruses			
Antibiotics are effective in the treatment of cold or flu			
Overuse of antibiotics makes them become ineffective			
A withdrawal period has to be strictly observed in treated poultry before any poultry product is passed as fit for human consumption			
A withdrawal period does not have to be observed for milking cows treated with antibiotics such as penicillin before milk can be consumed			

29. Do you believe as a Medical Laboratory Scientist, you have a role to play in preventing public health threats posed by antibiotic resistance?

• Yes
• No
• Don’t know

30. Please let us know any comment, suggestion, information, or opinion on the topics of this questionnaire. (Optional)

